# An integrated network of microRNA and gene expression in ovarian cancer

**DOI:** 10.1186/1471-2105-16-S5-S5

**Published:** 2015-03-18

**Authors:** Andrew Quitadamo, Lu Tian, Benika Hall, Xinghua Shi

**Affiliations:** 1 Department of Bioinformatics and Genomics, University of North Carolina at Charlotte, University City Blvd, Charlotte, NC, USA

**Keywords:** microRNA, ovarian cancer, cancer genomics, integrated network, expression Quantitative Trait Loci (eQTL) analysis

## Abstract

**Background:**

Ovarian cancer is a deadly female reproductive cancer. Understanding the biological mechanisms underlying ovarian cancer could help lead to quicker and more accurate diagnosis and more effective treatments. Both changes in microRNA(miRNA) expression and miRNA/mRNA dysregulation have been associated with ovarian cancer. With the availability of whole-genome miRNA and mRNA sequencing we now have new potentials to study these associations. In this study, we performed a comprehensive analysis of miRNA and mRNA expression in ovarian cancer using an integrative network approach combined with association analysis.

**Results:**

We developed an integrative approach to construct a network that illustrates the complex interplay among miRNA and gene expression from a systems perspective. Our method is composed of expanding networks from eQTL associations, building network associations in eQTL analysis, and then combine the networks into an integrated network. This integrated network takes account of miRNA expression quantitative trait loci (eQTL) associations, miRNAs and their targets, protein-protein interactions, co-expressions among miRNAs and genes respectively. Applied to the ovarian cancer data set from The Cancer Genome Atlas (TCGA), we created an integrated network with 167 nodes containing 108 miRNA-target interactions and 145 from protein-protein interactions, starting from 44 initial eQTLs. This integrated network encompassed 26 genes and 14 miRNAs associated with cancer. In particular, 11 genes and 12 miRNAs in the integrated network are associated with ovarian cancer.

**Conclusion:**

We demonstrated an integrated network approach that integrates multiple data sources at a systems level. We applied this approach to the TCGA ovarian cancer dataset, and constructed a network that provided a more inclusive view of miRNA and gene expression in ovarian cancer. This network included four separate types of interactions among miRNAs and genes. Simply analyzing each interaction component in isolation, such as the eQTL associations, the miRNA-target interactions or the protein-protein interactions, would create a much more limited network than the integrated one.

## Background

Ovarian cancer is the deadliest reproductive cancer in women, accounting for 5% of female cancer deaths. It is estimated that there will be 21,980 new cases and 14,270 deaths from ovarian cancer in 2014 [1]. The overall 5 year relative survival is less than 44%, but when diagnosed in the earliest stages the 5 year survival is over 90%. [2]. Therefore, a better understanding of the biological mechanisms of ovarian cancer is crucial to create earlier diagnosis and more effective treatment.

MicroRNAs (miRNAs) are small (~22 nucleotides) non-coding RNAs that regulate gene expression by targeting complementary mRNA, which triggers a translational blockade or degradation[3]. miRNAs have been increasingly recognized as an important participant of gene regulation activity [4] that affect various cellular processes [5] and contribute to disease pathogenesis in a wide variety of diseases [6] including cancer [7]. Changes in miRNA expression and miRNA dysregulation have been found in cancer, including ovarian cancer[8-13]. In another recent study, miRNAs were described as regulators of tumor phenotype [14]. Early studies show that miRNA expression profiles can be used to classify cancer phenotypes [15]. A study by The Cancer Genome Atlas (TCGA) found three miRNA subtypes in high grade serous ovarian cancer[16]. By exploiting the miRNA expression profiles and their effects on gene expression and other phenotypes in cancer, more profound markers for prognosis can be identified and utilized.

Genetic variation has also been determined to have functional effects on cancer through miRNA expression as well. Various studies have indicated the genetic variants such as single nucleotide polymorphisms (SNPs) are associated with cancer risk in individuals [17]. Particularly, eQTL analysis [18-25] provides a powerful tool to study how genetic variation affects quantitative traits, including measurable phenotypes like gene or miRNA expression. For instance, miRNA eQTL analysis [6,26,27] has also been performed to find genetic loci that introduce differences in miRNA expression.

In general, eQTL analysis analyzes the effect of genetic variation on gene expression by utilizing correlation or regression analysis between the genotypes of a genetic variant and a particular trait [25]. Such analysis assumes that genetic variants are independent, and that the expressions of different genes are independent as well. Nonetheless, many genetic variants are correlated, and the genes can be co-regulated as well. To address the correlation or interactions among these genetic variants or genes, new machine learning methods such as multi-task learning methods [28,29] have been designed to consider all the genes during regression while considering the network structure among genetic variants and genes [30-32]. New methods have also been developed to seek for other relationships, such as redundancy among eQTLs [33] where multiple genetic variants have the same effect on a particular trait and are thus exchangeable.

However, it is still unclear to what extent that changes in miRNA expression, an epigenetic marker, affect gene expression and bring about fluctuations in biological pathways and networks in ovarian cancer. It has recently been determined that some miRNAs promote epithelial-to-mesenchymal transition (EMT) and in turn, EMT transition promotes cell mobility and invasion of cancerous cells [14]. In efforts to understand such complex effects of miRNA expression on gene expression, we develop an integrated network approach that combines expression quantitative trait locus (eQTL) analysis with network analysis using various types of relationships and interactions among miRNAs and genes.

In order to understand the effects discussed above, we perform a genome wide eQTL analysis between miRNA expression and gene expression in ovarian cancer. The suggested method can help identify a set of miRNA eQTLs that have significant impact on gene expression. Using these miRNA eQTLs and their perturbed genes as seed nodes for network expansion, we created an integrated network that utilizes multiple types of networks such as miRNA targets and protein-protein interactions. In parallel, we used another graph guided machine learning model for eQTL analysis that incorporates correlations among miRNAs and genes simultaneously [30]. With these various types of relationships combined, we are able to generate an integrated network that not only captures different types of interactions among these miRNAs and genes, but also includes important cancer genes embedded in a network view. Such an integrative approach thus generates insightful results that could shed light on the interaction of miRNA, gene expression and their contribution to the development and progression of ovarian cancer.

## Methods

We developed an integrative approach that combines the strength of eQTL analysis with network analysis, toward the goal of building an integrated network to understand the relationship between miRNA expression and gene expression. The integrated network is composed of various types of relationships among miRNAs and genes, including miRNA-gene eQTL associations, miRNAs and their targets, protein-protein interactions, and correlation networks of both miRNA and gene expression.

**Figure 1:**
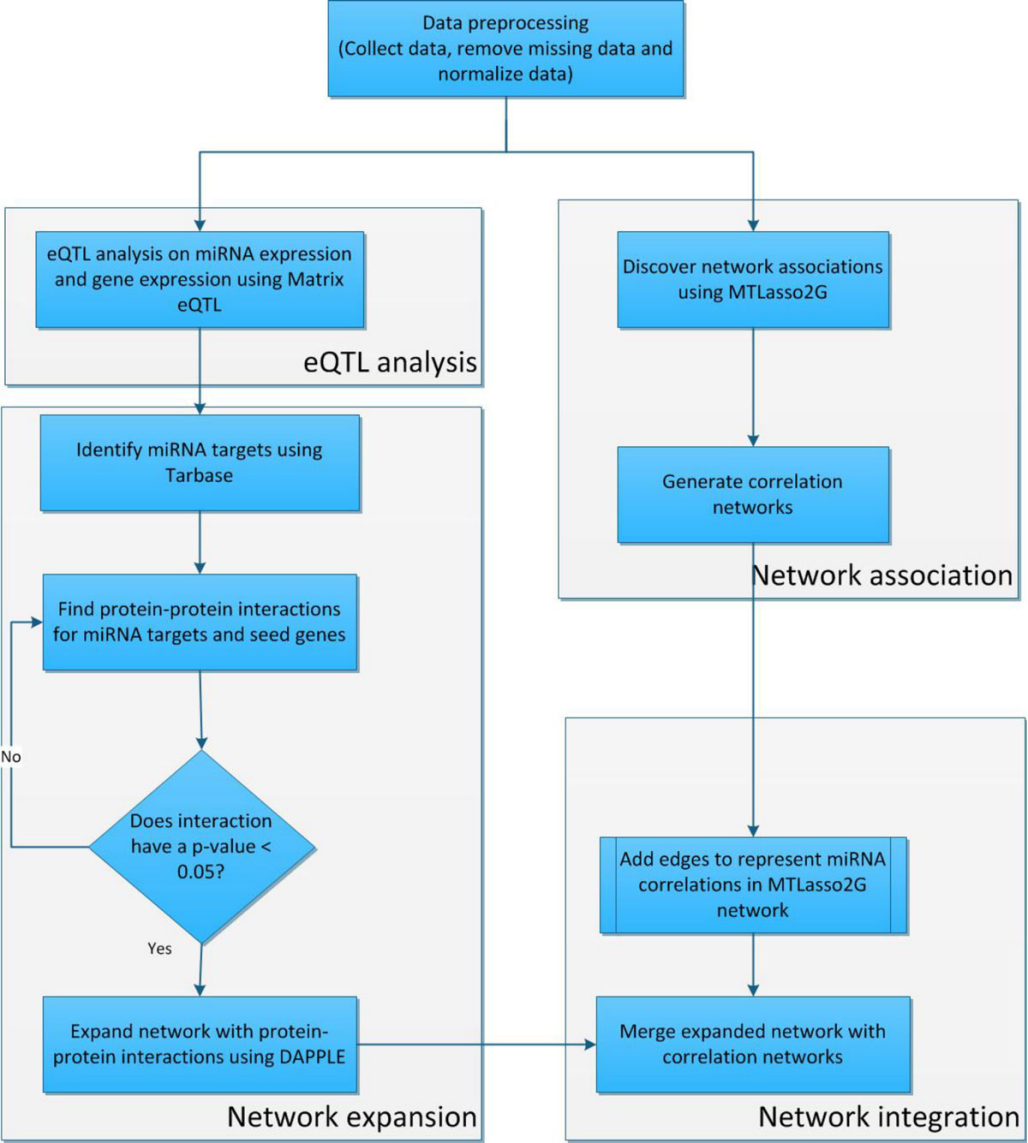
**The overall workflow of our integrative approach**. The workflow to create the integrated network consisted of four phases; eQTL analysis, network association, network expansion and network integration. We began with data pre-processing in which we collected data and normalized it. The first phase of the workflow started with an eQTL analysis between miRNA and gene expression data using Matrix eQTL. Once the eQTL analysis was complete, we proceeded to the network expansion phase. In the network expansion phase, we used TarBase to identify miRNA targets based on the eQTLs found in Matrix eQTL. Next, we found protein-protein interactions for the identified targets. To select the most confident interactions, we chose the interactions with a p-value ≤ 0.05. Then we expanded the network with the selected protein-protein interactions using DAPPLE, creating the first integrated network. Simultaneously, we used MtLasso2G to discover network associations between miRNAs, creating miRNA correlation networks. We then combined the eQTL expanded network with the correlation networks to create the final integrated network.

The overall workflow of our integrative approach is illustrated in Figure 1. Our approach consisted of five phases, including data preprocessing, eQTL analysis, network association, network expansion and network integration. Prior to analysis, we performed data pre-processing in which, we collected and normalized the data. The next phase of our workflow was the eQTL analysis between miRNA and gene expression. Here, we used Matrix eQTL to perform the analysis. In the network expansion phase, we used TarBase [34] to identify targets based on the eQTLs generated from Matrix eQTL [35]. Next, we found protein-protein interactions connected with the identified miRNA targets. In order to select the most confident interactions, we chose the interactions with a p-value ≤ 0.05. From there, we expanded the network using the selected protein-protein interactions using DAPPLE [36], generating the initial integrated network. Simultaneously, in the network association phase we used MtLasso2G [30] to discover network associations between miRNAs to build miRNA correlation networks. Finally, we merged the eQTL expanded network and the miRNA correlation networks to create the integrated network.

### Data preprocessing

We downloaded miRNA isoform and mRNA expression data for 480 samples of serous ovarian cancer from the TCGA data portal [37]. Both data sets were generated on the Illumina HiSeq platform. To pre-process the data, we removed samples with missing data and extracted quantifications for 183 miRNAs and 13,536 genes. To reduce the variation between samples, we used between-sample quantile normalization to normalize both the miRNA and gene expression data.

### Network expansion

To help exploit the relationships between miRNA and gene expression, we performed an eQTL analysis between the miRNA expression and gene expression profiles. The eQTL analysis was performed using Matrix eQTL, an R package [35]. Matrix eQTL was chosen because of its efficient computational time with large datasets [35] and its proven efficiency in many large studies [22,23]. We performed *cis *eQTL analysis, whereby we only tested miRNA and gene pairs that were within 1 MB of each other. To test for associations between the miRNA and genes, we used a linear regression model provided by Matrix eQTL. To correct for multiple comparisons, we utilized a multi-test correction based on false discovery rate (FDR) and chose the significant eQTLs with a FDR cutoff at < 0.01 for further analysis.

Using the eQTLs identified in Matrix eQTL as seed nodes for an initial network, we searched for linked targets of the miRNAs to expand the network. In order to identify miRNA targets, we used TarBase to generate a list of the miRNAs and their corresponding gene targets[34]. TarBase is a manually curated database of all experimentally verified miRNA targets. In generating the list, we discovered that some miRNAs may have multiple designated names associated with the 3' untranslated region (UTR) and the 5' untranslated region. In this case, we used both variations to keep it consistent with our data. For example, if there was an ambiguous miRNA name used in the TarBase search, it was replaced by both the -3' and -5' version of the name.

We used the miRNA target genes gathered from TarBase, in combination with the perturbed genes from the eQTLs, to further extend the network using protein-protein interactions. We chose the Disease Associated Protein-Protein Link Evaluator (DAPPLE)[36] for this step of network expansion since it's been well evaluated in finding candidate genes through expanding networks on signals from genome wide association studies [36,38,39]. By using DAPPLE on the miRNA target genes and the miRNA perturbed genes in the eQTL associations, we were able to find protein-protein interactions for miRNA targets and the miRNA eQTL perturbed genes. To perform the link evaluation, DAPPLE uses a statistical model to extend a network from an input seed gene list. The protein-protein interactions were derived from the InWeb database [40].

At each network expansion step, DAPPLE selects a new edge from the existing node set with the highest probability based on its significance against random permutations of the protein-protein interaction network. During the permutations, the network topology is conserved. For our analysis, we performed 1000 random permutations of the network. As part of our selection criteria, we only selected the interactions that contained a seed gene with a corrected p-value of ≤ 0.05. This allowed us to keep those interactions with a high-confidence for further analysis. We then overlapped the selected protein-protein interactions, the eQTLs and the miRNA-target interactions to create an initial integrated network.

### Network associations

In parallel with the network expansion, we used a two-graph guided multitask Lasso (MtLasso2G) to perform a second eQTL analysis [30]. MtLasso2G is an orthologous approach capable of determining network associations. Unlike Matrix eQTL, which we used to perform linear regression for each pair of miRNA and gene independently for eQTL analysis, MtLasso2G uses a sparse learning model that incorporates the relationships among miRNAs and genes. Here, we used a correlation expression network among miRNAs, and another co-expressed network among genes that potentially captured the structure of co-regulation networks. The MtLasso2G model accomplished eQTL analysis by adding two regularization terms based on the two graphs on features (i.e. miRNA expression) and labels (i.e. gene expression), which are encoded as miRNA co-expression network and gene co-expression network in our analysis respectively. The model can then be learned by minimizing the following objective function in a linear system:

$$\underset{\beta }{\text{min}}\;||Y-XB|{|}_{F}^{2}+\lambda ||\beta |{|}_{1}$$

(1) $$+\gamma_{1}\underset{e_{m,l}\in E_{1}}{\sum }w\left(e_{m,l}\right)\overset{J}{\underset{j=1}{\sum }}|b_{jm}-Sign\left(r_{m,l}\right)b_{jl}|$$

(2) $$+\gamma_{2}\underset{e_{f,g}\in E_{2}}{\sum }w\left(e_{f,g}\right)\overset{K}{\underset{k=1}{\sum }}|b_{fk}-Sign\left(r_{f,g}\right)b_{gk}|.$$

The two graphs, *G*_1 _= (*V*_1_, *E*_1_) and *G*_2 _= (*V*_2_, *E*_2_) represent the correlation networks among genes and miRNAs respectively. *V*_1 _and *V*_2 _are the nodes in *G*_1 _and *G*_2_. *E*_1 _and *E*_2 _are the edges representing the correlations among the corresponding vertices. *w*(·) is the edge weight. Individual edges in *G*_1 _and *G*_2 _are represented as *e_m,l _*and *e_f,g _*respectively. The correlation between *y^m ^*and *y^l ^*is denoted as *r_m,l_*, and *r_f,g _*denotes the correlation between *y^f ^*and *y^g^*. The learned *β *matrix from the model represents the association between each feature and label. Those nonzero terms stand for the identified eQTLs. As the MtLasso2G model captures the correlation structures among the miRNAs and genes, it produces not only a set of eQTLs, but also network associations. These network associations include a subnetwork capturing miRNA correlations, and another subnetwork representing the correlations among the perturbed genes.

### Network integration

We finally created an integrated network by merging the expanded network extended using DAPPLE, and the network associations generated from the MtLasso2G model. Specifically, we added representations of the correlation graphs from MtLasso2G to the expanded network from DAPPLE as following. If one miRNA was present in the current integrated network, we added highly correlated miRNAs. If both genes were already present in the current integrated network, we added an edge to the network.

The edges between miRNAs and perturbed genes from network associations in MtLasso2G are added to the integrated network as well. By merging the networks generated from DAPPLE and MtLasso2G as stated above, we created the final integrated network which was then visualized using Cytoscape[41]. Integrating the networks in this manner allowed us to conserve many of the pre-determined relationships found early on in the analysis.

## Results and discussion

**Figure 2:**
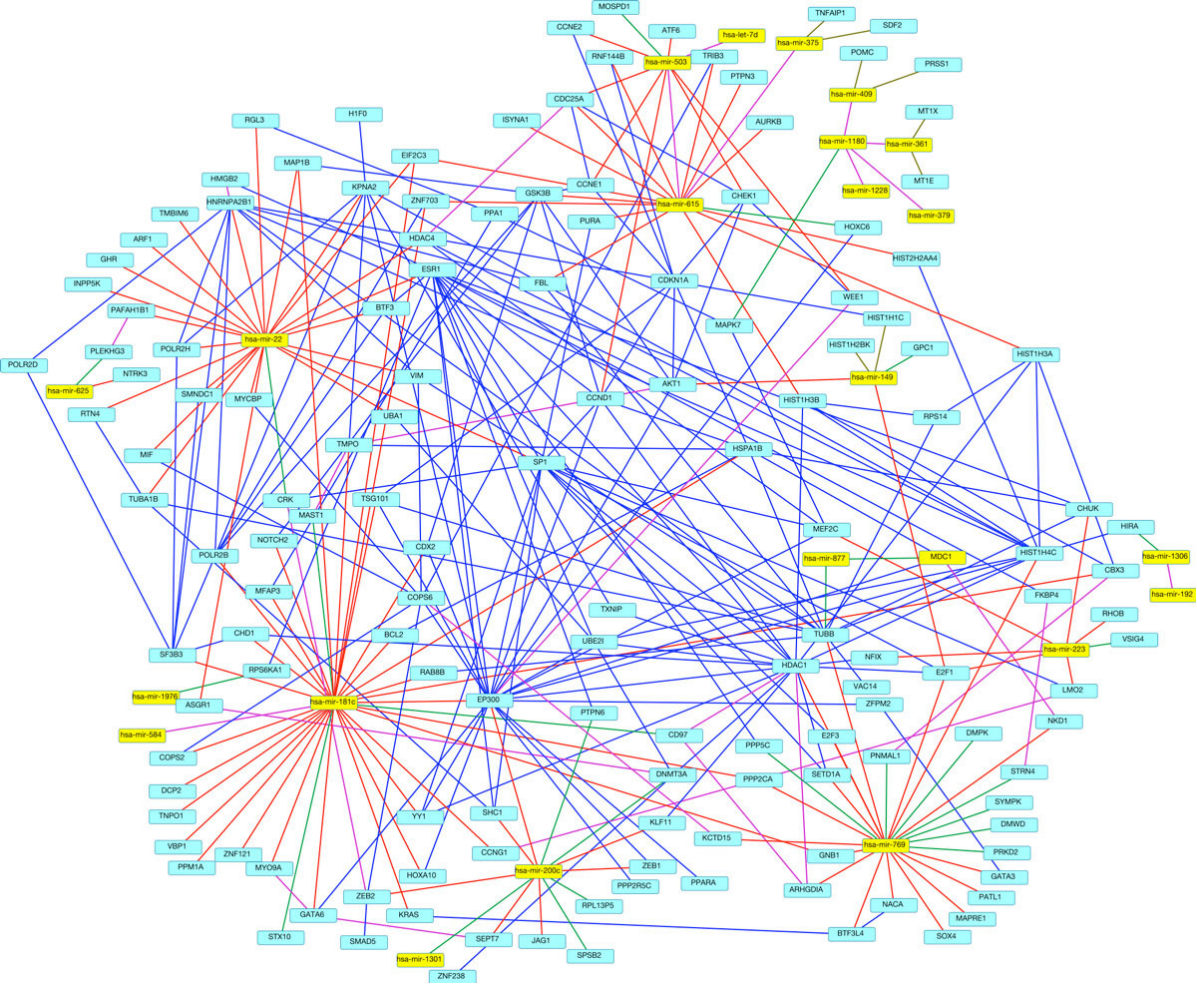
**The integrated network incorporating multiple data sources**. This network incorporated eQTLs, protein-protein interactions from DAPPLE, and miRNA-target interactions from TarBase. miRNAs are shown in yellow nodes, and genes are shown in light blue nodes. eQTLs are shown as green edges, protein-protein interactions are shown as blue edges, miRNA-target interactions are shown as red edges, MtLasso2G correlation interactions are in purple, and MtLasso2G eQTLs are in gold.

Our approach generates an integrated network (Figure 2) that incorporates the comprehensive relationship between miRNA and gene expression. We create this network from multiple sources of data and analysis including miRNA eQTL and associated genes, miRNAs and their target genes, protein-protein interactions among miRNA eQTL perturbed genes and miRNA target genes. This network thus captures the complexity of the interplay among miRNAs and genes in ovarian cancer, and point to a list of candidate miRNAs and genes for future studies.

Our method is scalable and applicable to integrating many genomic datasets. Here, as a demonstration of our approach, we apply it to the miRNA and gene expression data in ovarian cancer from TCGA [37]. After pre-processing, we use the expression of 183 miRNAs, and the expression of 13,536 genes from 480 samples.

**Figure 3:**
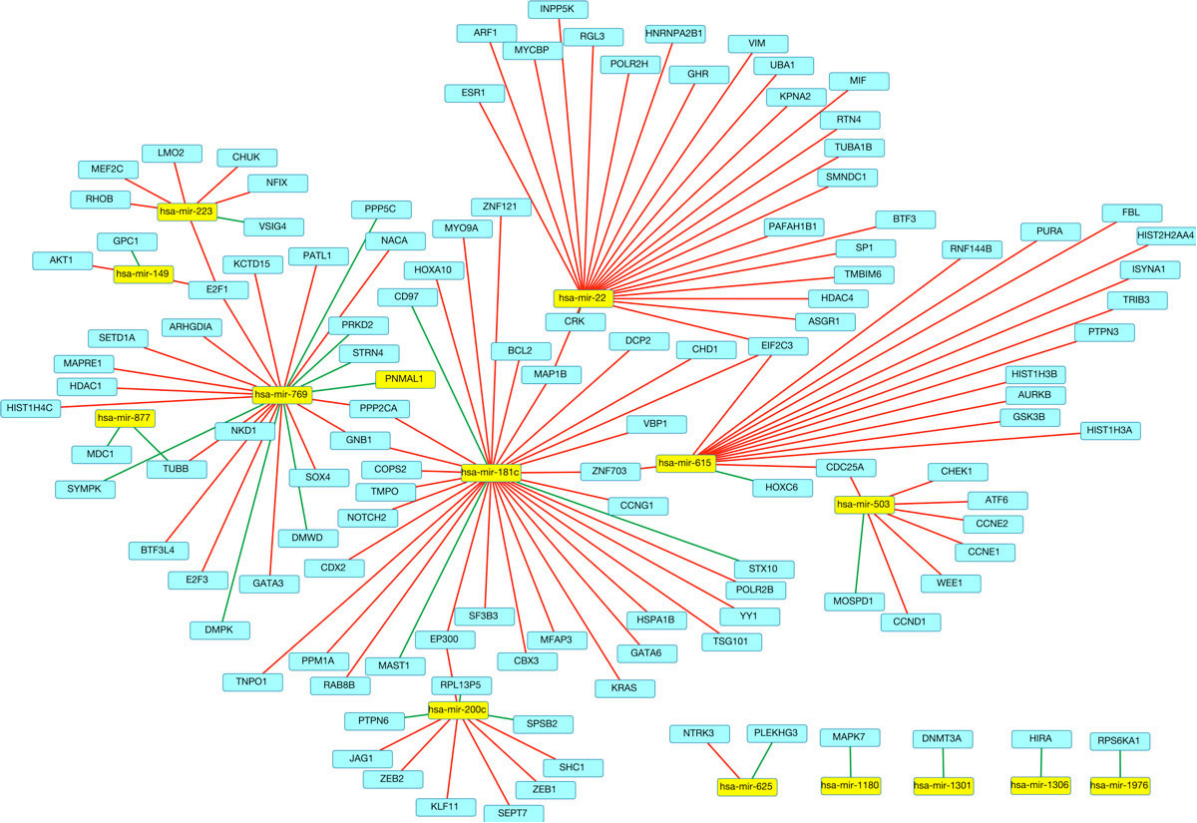
**The miRNA-target and eQTL network**. This network contains the eQTLs as well as the target genes from TarBase for the miRNAs. The initial integrated network is formed when the protein-protein interactions for the genes in this network are added.

We identified 44 miRNA eQTLs with a FDR < 0.01 using Matrix eQTL. There were 16 unique miRNAs, and 44 unique genes represented in the eQTLs. We found 310 unique target genes using TarBase, of which 244 could be used as inputs for DAPPLE. Figure 3 shows the miRNA-taget and eQTL network. There were 236 direct connections found using DAPPLE, and 145 that contained a seed gene with a corrected p-value of <0.05. We added 9 correlated miRNAs, and 18 correlated gene edges to the network based on the MtLasso2G correlation graph.

With MtLasso2G, we found 48 eQTLs, and were able to add 8 of them to the network, composed of the miRNA-target interactions found in TarBase, the eQTLs from Matrix eQTL, and the MtLasso2G correlations. This process created the final integrated network including 167 nodes and 277 edges shown in Figure 2. Table 1 summarizes the number of cancer genes found in each part of the analysis. Of the 145 gene nodes in this integrated network, 26 were found to be cancer related, and 11 of those were associated with ovarian cancer[42,43], as illustrated in Table 2.

**Table 1:** Cancer associated genes in the network components and the integrated network.

	DAPPLE Extended Network	miRNA Targets	MtLasso2G Network	Integrated Network
Genes	93	97	39	167

Cancer Genes[42]	20	22	6	26

Ovarian Cancer[43]	11	10	4	11

**Table 2:** List of cancer and ovarian cancer associated genes in the integrated network.

Genes Associated with Cancer	Genes Associated with Ovarian Cancer
AKT1	AKT1
BCL2	BCL2
CCND1	CCND1
CD97	CDC25A
CDC25A	CDKN1A
CDKN1A	E2F1
CDX2	E2F3
COPS2	EP300
E2F1	GATA6
E2F3	KRAS
EP300	SHC1
GATA6	
GSK3B	
JAG1	
KRAS	
LMO2	
MAPK7	
MAPRE1	
MIF	
NOTCH2	
NTRK3	
RHOB	
RPS6KA1	
RTN4	
SHC1	
SOX4	

**Figure 4:**
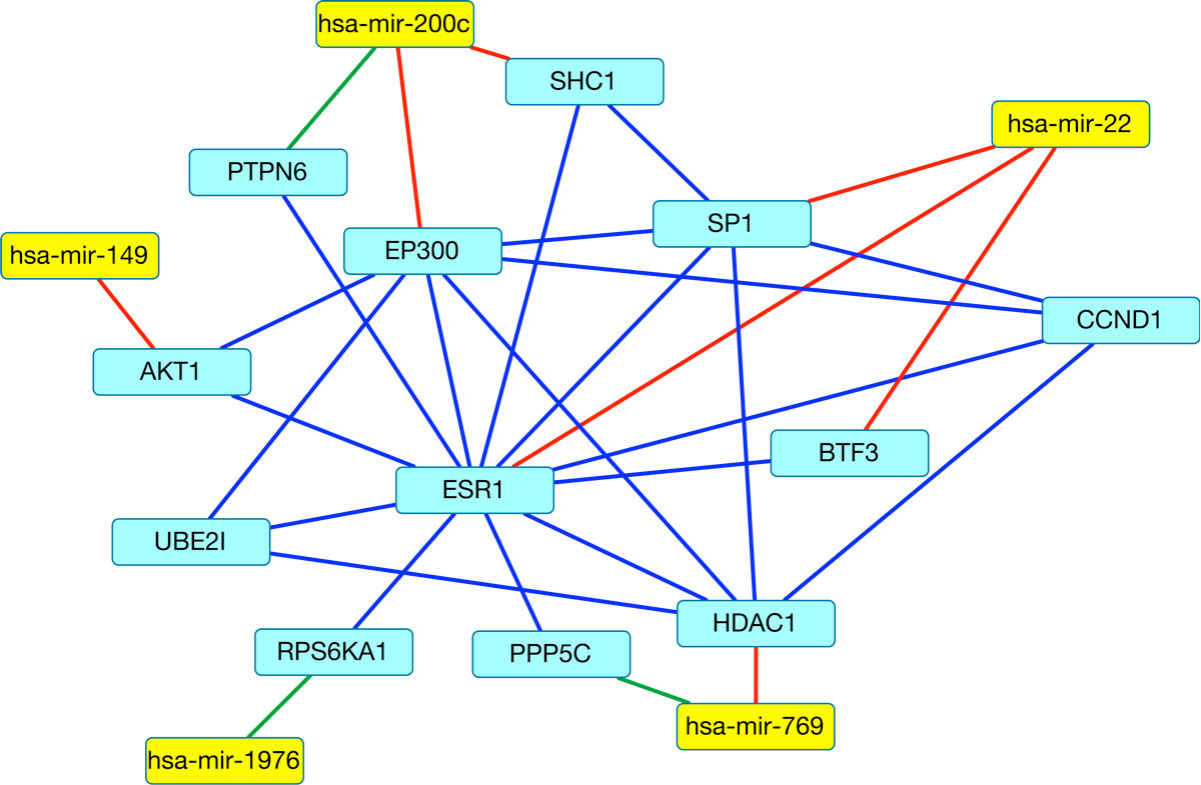
**A subnetwork illustrating multiple data sources for network construction**. This subnetwork illustrates how the multiple data sources are involved in the integrated network. If the network just contained the eQTLs in green, and the miRNA-target interactions in red, most of the connections would not exist. Adding the protein-protein interactions of the genes, allows us to visualize potential downstream effects.

One of the genes found in the integrated network, *AKT *1, has been implicated in tumorigenesis, specifically in ovarian cancer[44,45]. In our analysis *AT K*1 was found in the DAPPLE extended network, the miRNA-target network and the MtLasso2G gene-correlation graph. Figure 4 shows a subnetwork involving *AKT *1 of our integrated network. This subnetwork involves 5 miRNAs and 12 genes, connected by 31 edges. Three of the edges are eQTLs, and six edges were miRNA-target interactions. Another gene in the subnetwork was *ESR*1, which has been associated with breast cancer, and *ESR*1 expression has been suggested as a predictor of ovarian cancer survival[46-48].

Interestingly, in addition to capturing known ovarian cancer genes, this subnetwork provides a set of miRNAs and genes interacting with these known cancer genes. We propose that these interactive miRNAs and genes offer a new list of potential miRNA and gene markers for tumorigenesis, particularly in ovarian cancer.

Another interesting gene in the integrated network is *E*2*F *1 (Figure 2), which was targeted by three miRNAs. *E*2*F *1 has been studied as a predictor of drug resistance in ovarian cancer, and the down regulation of *E*2*F *1 has been shown to inhibit growth of ovarian cancer [49,50]. Therefore, *E*2*F *1 and their interactive partners are worthy of follow up studies for tumorigenesis as well.

We then analyzed the role of the miRNA eQTLs in different diseases including cancer and particularly ovarian cancer. Using multiple datasets (HMDD2, miRCancer, Phenomir, miRdSNP, miR2Disease, miRPD and Marchini) [51-57], we found that 19 of the 22 miRNAs have been implicated in some type of disease. Of these miRNAs, 14 were associated with a form of cancer, and 12 were specifically associated with ovarian cancer. Table 3 lists these cancer and ovarian cancer related miRNAs in our integrated network. Some of these miRNAs have demonstrated biological and clinical utility and could serve as disease biomarkers. For example, increased expression of hsa-miR-200c, one of the miRNAs found in our analysis, has been associated with increased survival, decreased chance of relapse and increased sensitivity to paclitaxel, a chemotherapy drug[57-59]. hsa-mir-22 has been identified as an marker for both survival and recurrence of ovarian cancer[60]. hsa-mir-22 has also been suggested as an inhibitor of cell movement and invasion in ovarian cancer[61]. Both hsa-mir-22 and hsa-mir-200c are found in the subnetwork, with hsa-mir-22 targeting *ESR*1.

**Table 3:** Number and names of miRNAs that are disease associated, cancer associated and ovarian cancer associated respectively.

	miRNAs	Disease associated miRNA	Cancer associated miRNA	Ovarian Cancer associated miRNA
**Number**	**22**	**19**	**14**	**12**

Names	hsa-let-7d	hsa-mir-1180	hsa-mir-149	hsa-mir-149
	hsa-mir-1180	hsa-mir-1228	hsa-mir-181c	hsa-mir-181c
	hsa-mir-1228	hsa-mir-1301	hsa-mir-192	hsa-mir-192
	hsa-mir-1301	hsa-mir-149	hsa-mir-200c	hsa-mir-200c
	hsa-mir-1306	hsa-mir-181c	hsa-mir-22	hsa-mir-22
	hsa-mir-149	hsa-mir-192	hsa-mir-223	hsa-mir-223
	hsa-mir-181c	hsa-mir-200c	hsa-mir-361	hsa-mir-375
	hsa-mir-192	hsa-mir-22	hsa-mir-375	hsa-mir-379
	hsa-mir-1976	hsa-mir-223	hsa-mir-379	hsa-mir-503
	hsa-mir-200c	hsa-mir-361	hsa-mir-409	hsa-mir-584
	hsa-mir-22	hsa-mir-375	hsa-mir-503	hsa-mir-625
	hsa-mir-223	hsa-mir-379	hsa-mir-584	hsa-mir-877
	hsa-mir-361	hsa-mir-409	hsa-mir-625	
	hsa-mir-375	hsa-mir-503	hsa-mir-877	
	hsa-mir-379	hsa-mir-584		
	hsa-mir-409	hsa-mir-615		
	hsa-mir-503	hsa-mir-625		
	hsa-mir-584	hsa-mir-769		
	hsa-mir-615	hsa-mir-877		
	hsa-mir-625			
	hsa-mir-769			
	hsa-mir-877			

## Conclusions

Changes in miRNA and mRNA expression are known to be involved in both ovarian cancer development and progression. Pin-pointing the exact changes and the relationships that occur between them could lead to advances in how ovarian cancer is treated and diagnosed. Creating an integrated network involving eQTLs, miRNA targets, protein-protein interactions and correlation graphs is one way to explore these relationships. Integrating multiple data sources allows us to create a wider and more holistic view of the interactions in ovarian cancer. This can lead us to understanding more about the underlying mechanisms that affect progression in ovarian cancer.

Therefore, in this paper we developed a new method of constructing an integrated network by combining the strength of eQTL study and network analysis. As a demonstration, we applied our method to TCGA ovarian cancer data and constructed an integrated network of miRNA and gene interactions in ovarian cancer. Utilizing disparate data sources allowed us to find relationships that would not have been visible otherwise. The network information we incorporated included protein-protein interactions, eQTL associations and miRNA-target interactions. None of those interactions alone would have shown all of the connections, but combining all these network information together allows us to see the complex interplay between miRNAs and genes in ovarian cancer. Our integrated analysis found miRNAs and genes linked to cancer and specifically to ovarian cancer. Utilizing integrated approaches like this one, could lead to a more comprehensive understanding of the biology that drives ovarian cancer.

In this study we used multiple data sources including one miRNA target database (i.e. TarBase), a protein-protein interaction network (i.e. InWeb) and expression data from the TCGA data portal to construct an integrated network. Based on recent studies [15,17], it is known that miRNAs and genetic variation have a critical impact on cancer progression and to an extent, cancer phenotype. Our approach allows us to incorporate various data sources into an integrated network. The generated network contained miRNA to target gene relationships, eQTL associations, protein-protein interactions, the correlations or co-expressions among microRNAs and genes respectively.

In the future, we will extend our approach by incorporating multiple databases of a particular network type, such as the STRING database [62] for protein-protein interaction networks. We will also incorporate other types of network information. For example, we can include regulatory and signaling pathways accumulated in existing pathway databases. Using various data sources, we can exploit the relationships between different molecular components to help understand how ovarian cancer progresses. Although ovarian cancer is a very complex disease, our approach is a leading step for future investigative methods.

## Competing interests

The authors declare that they have no competing interests.

## Authors' contributions

AQ and XS contributed to the design of the study. AQ performed the implementation and analysis of the project. AQ, XS, LT and BH performed the interpretation of the results and wrote the manuscript. All the authors read and approved the final manuscript.

## Declarations

Publication costs for this article were funded by the senior author's institute.

This article has been published as part of *BMC Bioinformatics *Volume 16 Supplement 5, 2015: Selected articles from the 10th International Symposium on Bioinformatics Research and Applications (ISBRA-14): Bioinformatics. The full contents of the supplement are available online at http://www.biomedcentral.com/bmcbioinformatics/supplements/16/S5.
